# Quadratic Solid–Shell Finite Elements for Geometrically Nonlinear Analysis of Functionally Graded Material Plates

**DOI:** 10.3390/ma11061046

**Published:** 2018-06-20

**Authors:** Hocine Chalal, Farid Abed-Meraim

**Affiliations:** Laboratory LEM3, Université de Lorraine, CNRS, Arts et Métiers ParisTech, F-57000 Metz, France; hocine.chalal@ensam.eu

**Keywords:** quadratic solid–shell elements, finite elements, functionally graded materials, thin structures, geometrically nonlinear analysis

## Abstract

In the current contribution, prismatic and hexahedral quadratic solid–shell (SHB) finite elements are proposed for the geometrically nonlinear analysis of thin structures made of functionally graded material (FGM). The proposed SHB finite elements are developed within a purely 3D framework, with displacements as the only degrees of freedom. Also, the in-plane reduced-integration technique is combined with the assumed-strain method to alleviate various locking phenomena. Furthermore, an arbitrary number of integration points are placed along a special direction, which represents the thickness. The developed elements are coupled with functionally graded behavior for the modeling of thin FGM plates. To this end, the Young modulus of the FGM plate is assumed to vary gradually in the thickness direction, according to a volume fraction distribution. The resulting formulations are implemented into the quasi-static ABAQUS/Standard finite element software in the framework of large displacements and rotations. Popular nonlinear benchmark problems are considered to assess the performance and accuracy of the proposed SHB elements. Comparisons with reference solutions from the literature demonstrate the good capabilities of the developed SHB elements for the 3D simulation of thin FGM plates.

## 1. Introduction

Over the last decades, the concept of functionally graded materials (FGMs) has emerged, and FGMs were introduced in the industrial environment due to their excellent performance compared to conventional materials. This new class of materials was first introduced in 1984 by a Japanese research group, who made a new class of composite materials (i.e., FGMs) for aerospace applications dealing with very high temperature gradients [1,2]. These heterogeneous materials are made from several isotropic material constituents, which are usually ceramic and metal. Among the many advantages of FGMs, their mechanical and thermal properties change gradually and continuously from one surface to the other, which allows for overcoming delamination between interfaces as compared to conventional composite materials. In addition, FGMs can resist severe environment conditions (e.g., very high temperatures), while maintaining structural integrity.

Thin structures are widely used in the automotive industry, especially through sheet metal forming into automotive components. In this context, the finite element (FE) method is considered nowadays as a practical numerical tool for the simulation of thin structures. Traditionally, shell and solid elements are used in the simulation of linear and nonlinear problems. However, the simulation results require very fine meshes to obtain accurate solutions due to the various locking phenomena that are inherent to these elements, which lead to high computational costs. To overcome these issues, many researchers have devoted their works to the development of locking-free finite elements. More specifically, the technology of solid–shell elements has become an interesting alternative to traditional solid and shell elements for the efficient modeling of thin structures (see, e.g., [3,4,5,6,7,8]). Solid‒shell elements are based on a fully 3D formulation with only nodal displacements as degrees of freedom. They can be easily combined with various fully 3D constitutive models (e.g., orthotropic elastic behavior, plastic behavior), without any further assumptions, such as plane-stress assumptions. Based on the reduced-integration technique (see, e.g., [9]), they are often combined with advanced strategies to alleviate locking phenomena, such as the assumed-strain method (ASM) (see, e.g., [4]), the enhanced assumed strain (EAS) formulation (see, e.g., [10]), and the assumed natural strain (ANS) approach (see, e.g., [11]). Several FE formulations for the analysis of thin FGM structures have been developed in the literature. They can be classified into three main formulations: The shell-based FGM FE formulation, the solid-based FGM FE formulation, and the solid–shell-based FE formulation. The first formulation is considered as the most widely adopted approach for the modeling of 2D thin FGM structures. However, this approach requires specific kinematic assumptions in the FE formulation, such as the classical Kirchhoff plate theory, first and high-order shear theories, plane-stress assumption (see, e.g., [12,13,14,15]). The second approach is based on a 3D formulation of solid elements, in which a fully 3D elastic behavior for FGMs is adopted. In such an approach, some specific kinematic assumptions for thin plates, such as the classical Kirchhoff plate theory and the von Karman theory, are also adopted in the FE formulation (see, e.g., [16,17,18]). The third approach is based on the concept of solid–shell elements, which are combined with FGM behavior. Few works in the literature have investigated the behavior of thin FGM plates with this approach. Among them, the work of Zhang et al. [19], who investigated the piezo-thermo-elastic behavior of FGM shells with EAS-ANS solid–shell elements. Recently, Hajlaoui et al. [20,21] studied the buckling and nonlinear dynamic analysis of FGM shells using an EAS solid–shell element based on the first-order shear deformation concept.

In this work, quadratic prismatic and hexahedral shell-based (SHB) continuum elements, namely SHB15 and SHB20, respectively, are proposed for the modeling of thin FGM plates. SHB15 is a fifteen-node prismatic solid‒shell element with a user-defined number of through-thickness integration points, while SHB20 is a twenty-node hexahedral solid‒shell element with a user-defined number of through-thickness integration points. These solid‒shell elements have been first developed in the framework of isotropic elastic materials and small strains (see [22]), and recently coupled with anisotropic elastic–plastic behavior models within the framework of large strains for the modeling of sheet metal forming processes [23]. In this paper, the formulations of the quadratic SHB15 and SHB20 elements are combined with functionally graded behavior for the modeling of thin FGM plates. To achieve this, the elastic properties of the proposed elements are assumed to vary gradually in the thickness direction according to a power-law volume fraction. The resulting formulations are implemented into the quasi-static ABAQUS/Standard software. The performance of the proposed elements is assessed through the simulation of various nonlinear benchmark problems taken from the literature.

## 2. SHB15 and SHB20 Solid‒Shell Elements

### 2.1. Element Reference Geometries

**Figure 1:**
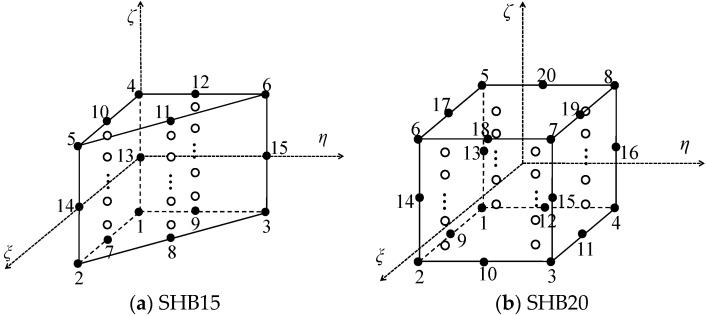
Reference geometry of (**a**) quadratic prismatic SHB15 element and (**b**) quadratic hexahedral SHB20 element, and position of the associated integration points.

The proposed SHB elements are based on a 3D formulation, with displacements as the only degrees of freedom. Figure 1 shows the reference geometry of the quadratic prismatic SHB15 and quadratic hexahedral SHB20 elements and the position of the associated integration points. Within the reference frame of each element, direction ζ represents the thickness, along which several integration points can be arranged.

### 2.2. Quadratic Approximation for the SHB Elements

Conventional quadratic interpolation functions for traditional continuum prismatic and hexahedral elements are used in the formulation of the SHB elements. According to this formulation, the spatial coordinates $x_{i}$ and the displacement field $u_{i}$ are approximated using the following interpolation functions:

(1) $$x_{i}=x_{iI}N_{I}(\xi ,\eta ,\zeta )=\sum_{I=1}^{K}x_{iI}N_{I}(\xi ,\eta ,\zeta ),$$

(2) $$u_{i}=d_{iI}N_{I}(\xi ,\eta ,\zeta )=\sum_{I=1}^{K}d_{iI}N_{I}(\xi ,\eta ,\zeta ),$$

where $d_{iI}$ are the nodal displacements, i=1, 2, 3 correspond to the spatial coordinate directions, and *I* varies from 1 to *K*, with *K* being the number of nodes per element, which is equal to 15 for the SHB15 element and 20 for the SHB20 element.

### 2.3. Strain Field and Gradient Operator

Using the above approximation for the displacement within the element, the linearized strain tensor ε can be derived as:

(3) $$\varepsilon_{ij}=\frac{1}{2}\left(u_{i,\;j}+u_{j,\;i}\right)=\frac{1}{2}\left(d_{iI}N_{I,\;j}+d_{jI}N_{I,\;i}\right).$$

By combining Equations (1) and (2) with the help of the interpolation functions, the nodal displacement vectors $d_{i}$ write:

(4) $$d_{i}=a_{0i}s+a_{1i}x_{1}+a_{2i}x_{2}+a_{3i}x_{3}+\sum_{\alpha }c_{\alpha i}h_{\alpha },\;i=1,\;2,\;3,$$

where $x_{i}^{T}=\left(x_{i1},\;x_{i2},\;x_{i3},\;\cdots ,\;x_{iK}\right)$ are the nodal coordinate vectors. In Equation (4), index α goes from 1 to 11 for the SHB15 element, and from 1 to 16 for the SHB20 element. In addition, vector $s^{T}=\left(1,\;1,\;\cdots ,\;1\right)$ has fifteen constant components in the case of the SHB15 element, and twenty constant components vector for the SHB20 element. Vectors $h_{\alpha }$ are composed of $h_{\alpha }$ functions, which are evaluated at the element nodes, and the full details of their expressions can be found in [23].

With the help of some well-known orthogonality conditions and of the Hallquist [24] vectors $b_{i}={\frac{\partial N}{\partial x_{i}}}_{|\xi =\eta =\zeta =0}$, where vector N contains the expressions of the interpolation functions $N_{I}$, the unknown constants $a_{ji}$ and $c_{\alpha i}$ in Equation (4) can be derived as:

(5) $$a_{ji}=b_{j}^{T}\cdot d_{i},\;c_{\alpha i}=\gamma_{\alpha }^{T}\cdot d_{i},$$

where the complete details on the expressions of vectors $\gamma_{\alpha }$ can be found in [22].

By introducing the discrete gradient operator B, the strain field in Equation (3) writes:

(6) $$\nabla_{s}\left(u\right)=\left[\begin{array}{c} u_{x,x}\\ u_{y,y}\\ u_{z,z}\\ u_{x,y}+u_{y,x}\\ u_{y,z}+u_{z,y}\\ u_{x,z}+u_{z,x} \end{array}\right]=B\cdot d=B\cdot \left[\begin{array}{l} d_{x}\\ d_{y}\\ d_{z} \end{array}\right],$$

where the expression of the discrete gradient operator B is:

(7) $$B=\left[\begin{array}{l} \begin{array}{ccc} b_{x}^{T}+h_{\alpha ,x}\gamma_{\alpha }^{T} & 0 & 0\\ 0 & b_{y}^{T}+h_{\alpha ,y}\gamma_{\alpha }^{T} & 0\\ 0 & 0 & b_{z}^{T}+h_{\alpha ,z}\gamma_{\alpha }^{T} \end{array}\\ \begin{array}{ccc} b_{y}^{T}+h_{\alpha ,y}\gamma_{\alpha }^{T} & b_{x}^{T}+h_{\alpha ,x}\gamma_{\alpha }^{T} & 0\\ 0 & b_{z}^{T}+h_{\alpha ,z}\gamma_{\alpha }^{T} & b_{y}^{T}+h_{\alpha ,y}\gamma_{\alpha }^{T}\\ b_{z}^{T}+h_{\alpha ,z}\gamma_{\alpha }^{T} & 0 & b_{x}^{T}+h_{\alpha ,x}\gamma_{\alpha }^{T} \end{array} \end{array}\right].$$

### 2.4. Hu–Washizu Variational Principle

The SHB solid‒shell elements are based on the assumed-strain method, which is derived from the simplified form of the Hu–Washizu variational principle [25]. In terms of assumed-strain rate $\dot{\bar{\varepsilon }}$, interpolated stress σ, nodal velocities $\dot{d}$, and external nodal forces $f^{ext}$, this principle writes

(8) $$\pi \left(\dot{\bar{\varepsilon }}\right)=\int_{\Omega_{e}}\delta {\dot{\bar{\varepsilon }}}^{T}\cdot \sigma \;d\Omega \;-\;\delta {\dot{d}}^{T}\cdot f^{ext}=0.$$

The assumed-strain rate is expressed in terms of the discrete gradient operator B as:

(9) $$\dot{\bar{\varepsilon }}\left(x,t\right)=B\cdot \dot{d}.$$

Substituting the expression of the assumed-strain rate given by Equation (9) into the variational principle (Equation (8)), the expressions of the stiffness matrix $K_{e}$ and the internal forces $f^{\mathrm{int}}$ for the SHB elements are

(10) $$K_{e}=\int_{\Omega_{e}}B^{T}\cdot C^{e}\left(\zeta \right)\cdot B\;d\Omega \;+\;K_{GEOM},$$

(11) $$f^{\mathrm{int}}=\int_{\Omega_{e}}B^{T}\cdot \sigma \;d\Omega ,$$

where $K_{GEOM}$ is the geometric stiffness matrix. As to the fourth-order tensor $C^{e}\left(\zeta \right)$, it describes the functionally graded elastic behavior of the FGM material. Its expression is given hereafter.

### 2.5. Description of Functionally Graded Elastic Behavior

**Figure 2:**
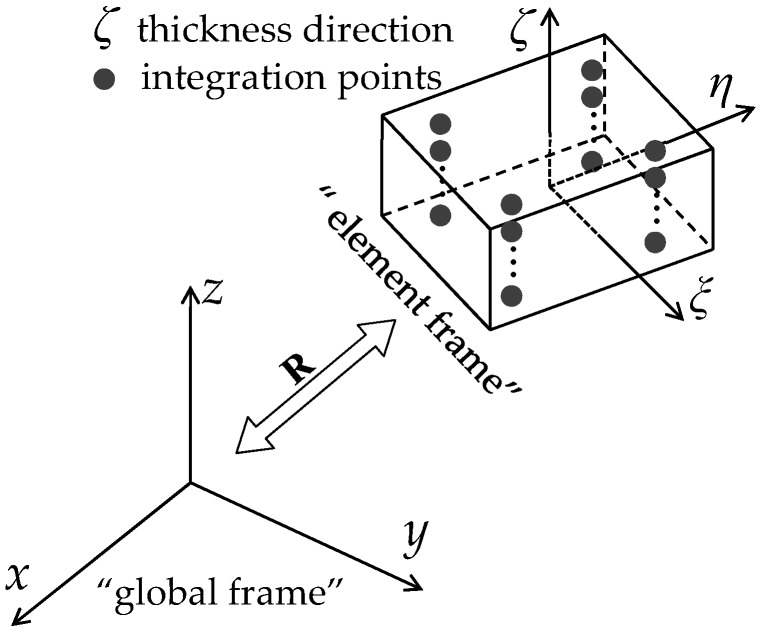
Element frame and global frame for the proposed SHB elements.

In the framework of large displacements and rotations, the formulation of the SHB elements requires the definition of a local frame with respect to the global coordinate system, as illustrated in Figure 2. The local frame, which is designated as the “element frame” in Figure 2, is defined for each element using the associated nodal coordinates. In such an element frame, where the ζ-coordinate represents the thickness direction, the fourth-order elasticity tensor $C^{e}\left(\zeta \right)$ for the FGM is specified.

**Figure 3:**
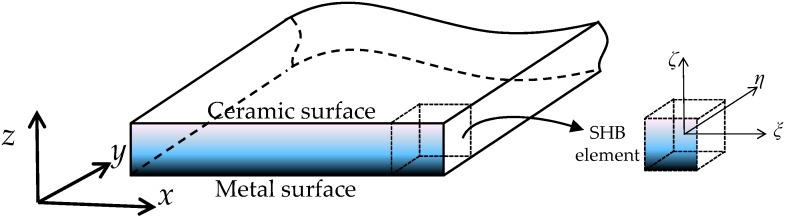
Schematic representation of the functionally graded thin plate.

**Figure 4:**
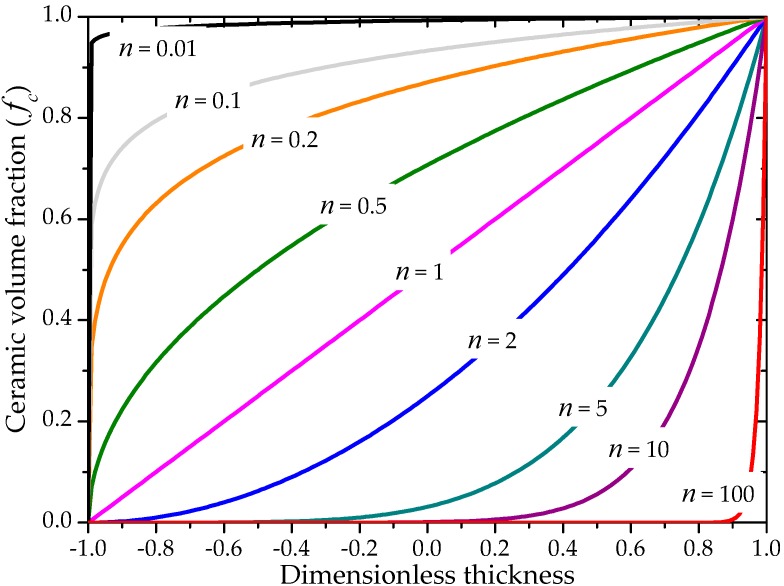
Volume fraction distribution of the ceramic phase as function of the power-law exponent *n*.

In this work, a two-phase FGM is considered, which consists of two constituent mixtures of ceramic and metal. The ceramic phase of the FGM can sustain very high temperature gradients, while the ductility of the metal phase prevents the onset of fracture due to the cyclic thermal loading. In such FGMs, the material at the bottom surface of the plate is fully metal and at the top surface of the plate is fully ceramic, as illustrated in Figure 3. Between these bottom and top surfaces, the elastic properties vary continuously through the thickness from metal to ceramic properties, respectively, according to a power-law volume fraction. The corresponding volume fractions for the ceramic phase $f_{c}$ and the metal phase $f_{m}$ are expressed as (see, e.g., [26,27]):

(12) $$f_{c}={\left(\frac{z}{t}+\frac{1}{2}\right)}^{n}\;\mathrm{and}\;f_{m}=1-f_{c},$$

where *n* is the power-law exponent, which is greater than or equal to zero, and z∈[−t/2, t/2], with t the thickness of the plate. For n=0, the material is fully ceramic, while when n→∞ the material is fully metal (see Figure 4).

For an isotropic elastic behavior, the constitutive equations are governed by the Hooke elasticity law, which is expressed by the following relationship:

(13) σ=λtr(ε)1+2με,

where 1 denotes the second-order unit tensor, λ and μ are the Lamé constants given by:

(14) $$\lambda =\frac{\nu E}{\left(1-2\nu \right)\left(1+\nu \right)}\;\mathrm{and}\;\mu =\frac{E}{2\left(1+\nu \right)},$$

with E and ν the Young modulus and the Poisson ratio, respectively.

For FGMs that are made of ceramic and metal constituents, it is commonly assumed that only the Young modulus E varies in the thickness direction, while the Poisson ratio ν is kept constant. Therefore, the constant Young modulus in Equation (14) is replaced by E(z), whose value evolves according to the following power-law distribution:

(15) $$E\left(z\right)=\left(E_{c}-E_{m}\right)f_{c}+E_{m},$$

where $E_{c}$ and $E_{m}$ are the Young modulus of the ceramic and metal, respectively.

## 3. Nonlinear Benchmark Problems

In this section, the performance of the proposed elements is assessed through the simulation of several popular nonlinear benchmark problems. The static ABAQUS/Standard solver has been used to solve the following static benchmark problems. More specifically, the classical Newton method is considered for most benchmark problems, aside from limit-point buckling problems for which the Riks arc-length method is used.

To accurately describe the variation of the Young modulus through the thickness of the FGM plates, only five integration points within a single element layer is used in the simulations. For each benchmark problem, the simulation results given by the proposed elements are compared to the reference solutions taken from the literature. In the subsequent simulations, it is worth noting that the elastic properties of the metal and ceramic constituents of the FGM plates do not reflect a real metallic or ceramic material. Indeed, the terms metal and ceramic are commonly used in the literature to emphasize the difference between the properties of the FGM constituents (see, e.g., [15,26,27]).

Regarding the meshes used in the simulations, the following mesh strategy is adopted: (N_1_ × N_2_) × N_3_ for the hexahedral SHB20 element, where N_1_ is the number of elements along the length, N_2_ is the number of elements along the width, and N_3_ is the number of elements along the thickness direction. As to the prismatic SHB15 element, the mesh strategy consists of (N_1_ × N_2_ × 2) × N_3_, due to the in-plane subdivision of a rectangular element into two triangles.

### 3.1. Cantilever Beam Sujected to End Shear Force

**Figure 5:**
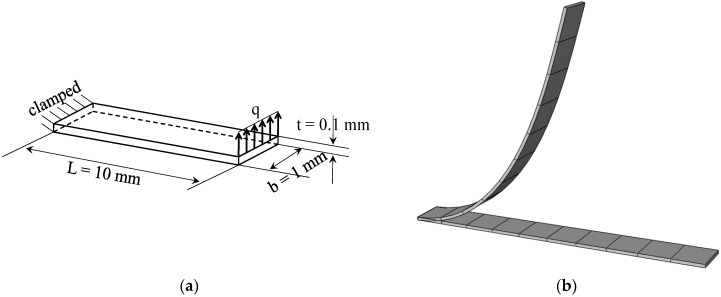
Cantilever beam: (**a**) geometry and (**b**) undeformed and deformed configurations.

**Figure 6:**
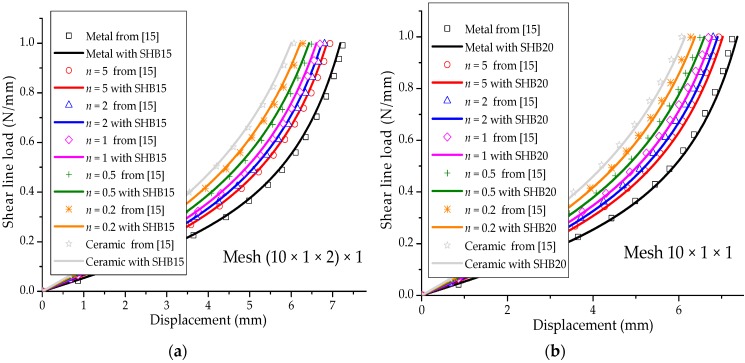
Load–deflection curves for the cantilever beam. (**a**) Prismatic SHB15 element; (**b**) hexahedral SHB20 element.

Figure 5a shows a simple cantilever FGM beam with a bending load at its free end. This is a classical popular benchmark problem, which has been widely considered in many works for the analysis of cantilever beams with isotropic material (see, e.g., [28,29]). The Poisson ratio of the FGM beam is assumed to be ν=0.3, while the Young modulus of the metal and ceramic constituents are $E_{m}=2.1\times 10^{5}\;\mathrm{Mpa}$ and $E_{c}=3.8\times 10^{5}\;\mathrm{Mpa}$, respectively. Figure 5b illustrates the final deformed shape of the cantilever beam with respect to its undeformed shape, as discretized with SHB20 elements, in the case of fully metallic material. Figure 6 shows the load–deflection curves obtained with the quadratic SHB elements, along with the reference solutions taken from [15], for various values of the power-law exponent *n.* One recalls that fully metallic material is obtained when n→∞, and fully ceramic material for n=0. Overall, the SHB elements show excellent agreement with the reference solutions corresponding to the various values of exponent *n*. More specifically, it can be observed that the bending behavior of the FGM beam lies between that of the fully ceramic and fully metal beam, which is consistent with the power-law distribution of the Young modulus in the thickness direction. Another advantage of the proposed SHB elements is that, using the same in-plane mesh discretization as in reference [15], only five integration points through the thickness are sufficient for the SHB elements, while ten integration points have been considered in [15] to simulate this benchmark problem.

### 3.2. Slit Annular Plate

**Figure 7:**
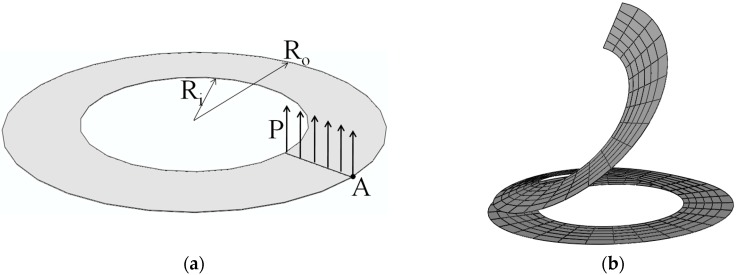
Slit annular plate: (**a**) geometry and (**b**) undeformed and deformed configurations.

**Figure 8:**
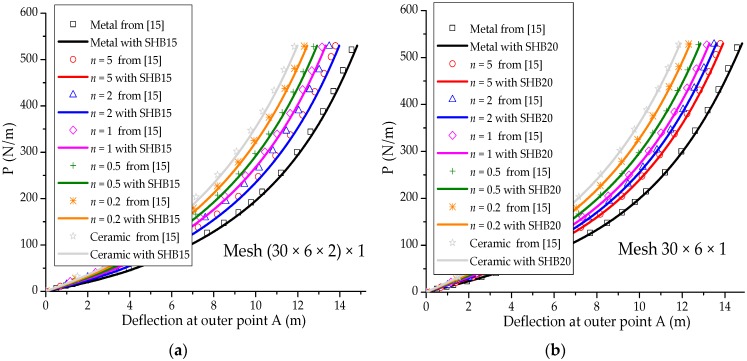
Load–deflection curves at the outer point A for the slit annular plate. (**a**) Prismatic SHB15 element; (**b**) hexahedral SHB20 element.

In this section, the well-known slit annular plate problem is considered (see, e.g., [29,30,31]). The annular plate is clamped at one end and loaded by a line shear force P, as illustrated in Figure 7a. The inner and outer radius of the annular plate are $R_{i}=6\;m$ and $R_{o}=10\;m$, respectively, while the thickness is t=0.03 m. The Poisson ratio of the annular plate is ν=0.3, while the Young modulus of the metal and ceramic constituents are $E_{m}=21\;\mathrm{Gpa}$ and $E_{c}=38\;\mathrm{Gpa}$, respectively. Figure 7b illustrates the undeformed and deformed shapes of the annular plate, as discretized with SHB20 elements, in the case of fully metallic material. Figure 8 reports the load–out-of-plane vertical deflection curves at the outer point A of the annular plate as obtained with the SHB elements, along with the reference solutions taken from [15]. One can observe that the SHB elements perform very well with respect to the reference solutions for all considered values of exponent *n*. Similar to the previous benchmark problem, the same in-plane mesh discretization as in [15] with only five integration points through the thickness has been adopted by the proposed SHB elements for this nonlinear test, while ten integration points have been considered in [15].

### 3.3. Clamped Square Plate under Pressure

**Figure 9:**
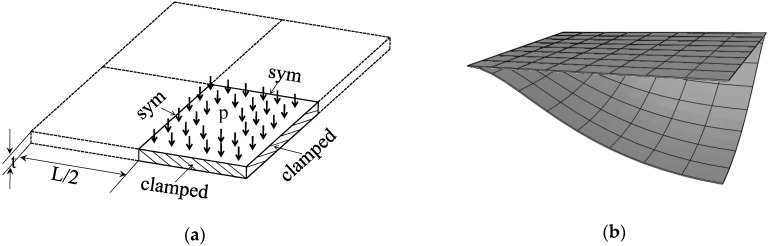
Clamped square plate: (**a**) geometry and (**b**) undeformed and deformed configurations.

**Figure 10:**
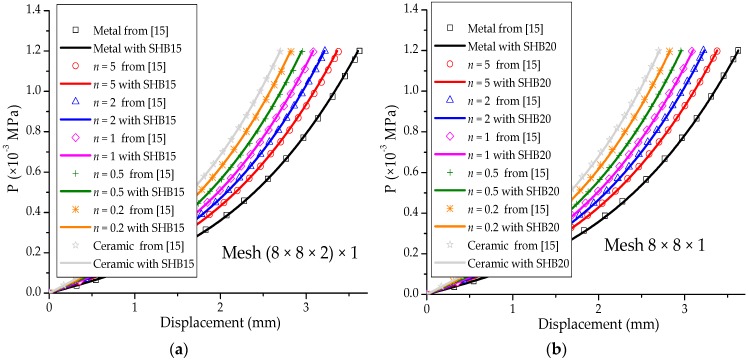
Load–deflection curves at the center point for the square plate. (**a**) Prismatic SHB15 element; (**b**) hexahedral SHB20 element.

Figure 9a illustrates a fully clamped square plate, which is loaded by a uniformly distributed pressure. The length and thickness of the square plate are L=1000 mm and t=2 mm, respectively. The Poisson ratio is ν=0.3, while the Young modulus of the metal and ceramic constituents are $E_{m}=2\times 10^{5}\;\mathrm{Mpa}$ and $E_{c}=3.8\times 10^{5}\;\mathrm{MPa}$, respectively. Considering the problem symmetry, a quarter of the plate is discretized. Figure 9b illustrates the undeformed and deformed shapes of the square plate, as discretized with SHB20 elements, in the case of fully metallic material. The pressure–displacement curves for the SHB elements (where the displacement is computed at the center of the plate), along with the reference solutions taken from [15], are all depicted in Figure 10. The results obtained with the SHB elements, by adopting only five integration points in the thickness direction and the same in-plane mesh discretization as in [15], are in excellent agreement with the reference solutions that required ten through-thickness integration points.

### 3.4. Hinged Cylindrical Roof

**Figure 11:**
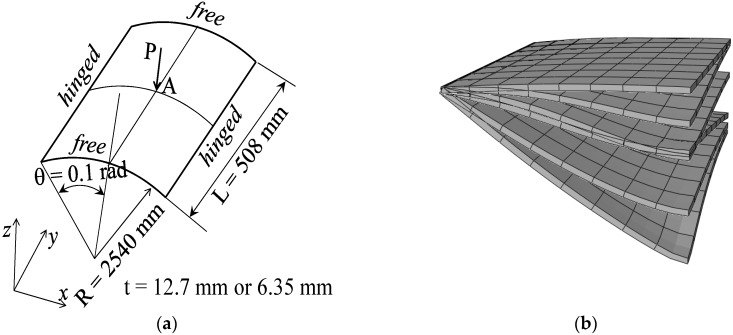
Hinged cylindrical roof: (**a**) geometry and (**b**) undeformed and deformed configurations.

**Figure 12:**
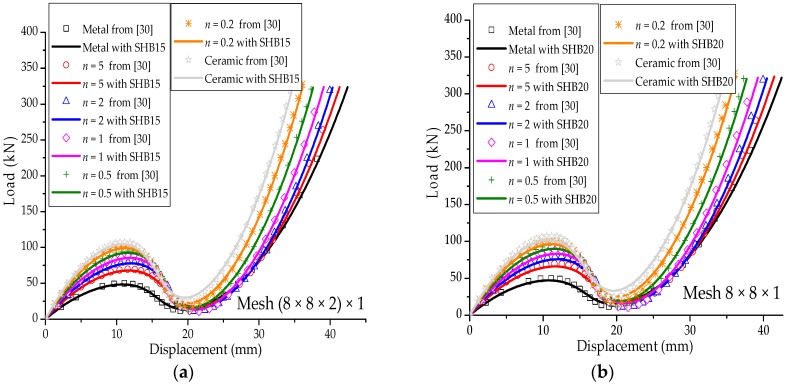
Deflection at the central point A under concentrated force for the thick hinged roof. (**a**) prismatic SHB15 element; (**b**) hexahedral SHB20 element.

**Figure 13:**
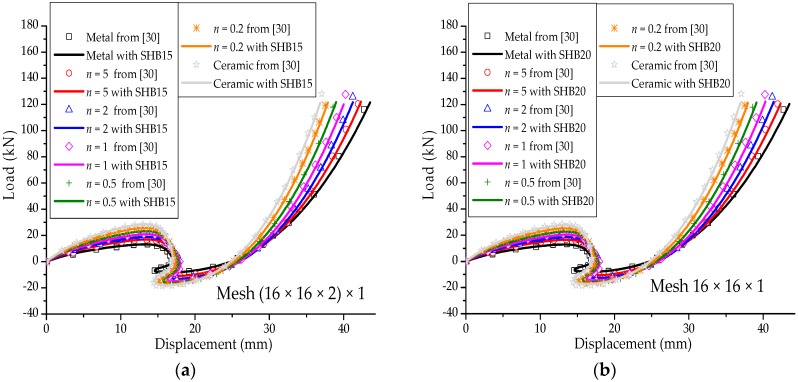
Deflection at the central point A under concentrated force for the thin hinged roof. (**a**) prismatic SHB15 element; (**b**) hexahedral SHB20 element.

Figure 11a shows a hinged cylindrical roof subjected to a concentrated force at its center. Two types of roofs are considered, thick and thin, with thicknesses t = 12.7 mm and t = 6.35 mm, respectively. Because this nonlinear benchmark test involves geometric-type instabilities (limit-point buckling), the Riks path-following method is used to follow the load–displacement curves beyond the limit points. The Poisson ratio of the cylindrical roof is ν=0.3, while the Young modulus of the metal and ceramic constituents are $E_{m}=70\times 10^{3}\;\mathrm{Mpa}$ and $E_{c}=151\times 10^{3}\;\mathrm{Mpa}$, respectively. Owing to the symmetry, only one quarter of the cylindrical roof is modeled. Figure 11b illustrates the undeformed and deformed shapes of the hinged cylindrical roof, as discretized with SHB20 elements, in the case of fully metallic material. The load–vertical displacement curves at the central point A of the thick and thin hinged cylindrical roofs are shown in Figure 12 and Figure 13, and compared with the reference solutions taken from [30]. From these figures, it can be seen that the results obtained with the proposed quadratic SHB elements are in good agreement with the reference solutions for the different values of exponent *n*, corresponding to different volume fractions (from fully metal to fully ceramic). More specifically, the snap-through and snap-back phenomena, which are typically exhibited in such limit-point buckling problems, are very well reproduced by the proposed SHB elements. Note that, for the thick roof (i.e., t = 12.7 mm), the converged solutions in Figure 12 are obtained by using a mesh of (8 × 8 × 2) × 1 in the case of prismatic SHB15 elements, and a mesh of 8 × 8 × 1 with hexahedral SHB20 elements. As to the thin roof (i.e., t = 6.35 mm), finer meshes of (16 × 16 × 2) × 1 for the prismatic SHB15 elements, and 16 × 16 × 1 for the hexahedral SHB20 elements are required to obtain converged results (see Figure 13). These mesh refinements are similar to those used by Sze et al. [29] for the thick and thin roof in the case of an isotropic material as well as for multilayered composite materials.

### 3.5. Pull-Out of an Open-Ended Cylinder

**Figure 14:**
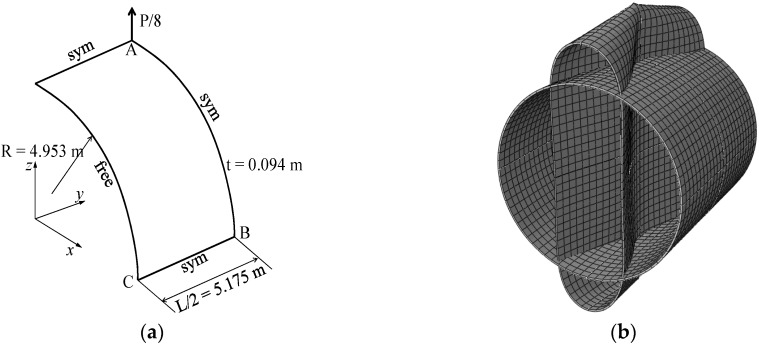
Pull-out of an open-ended cylinder: (**a**) geometry and (**b**) undeformed and deformed configurations.

**Figure 15:**
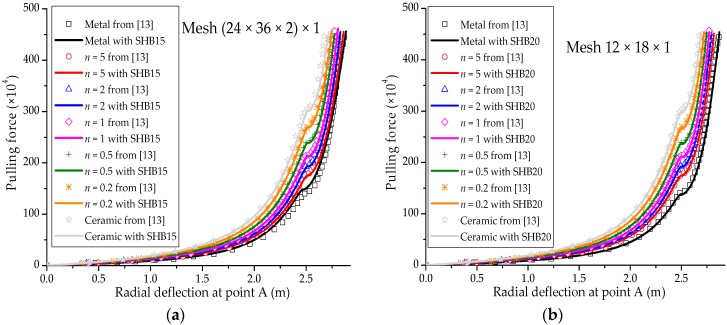
Radial displacement at point A under concentrated force for the open-ended cylinder. (**a**) Prismatic SHB15 element; (**b**) hexahedral SHB20 element.

**Figure 16:**
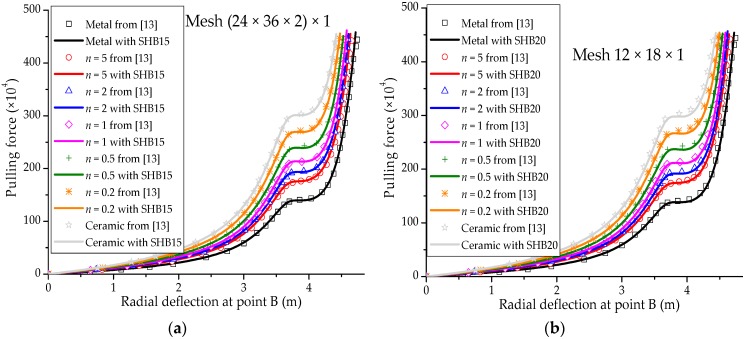
Radial displacement at point B under concentrated force for the open-ended cylinder. (**a**) prismatic SHB15 element; (**b**) hexahedral SHB20 element.

**Figure 17:**
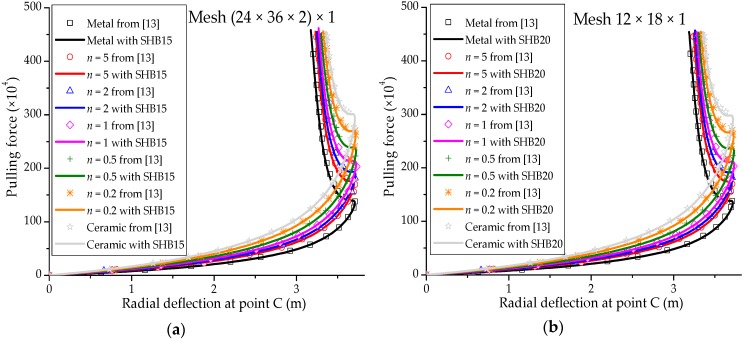
Radial displacement at point C under concentrated force for the open-ended cylinder. (**a**) prismatic SHB15 element; (**b**) hexahedral SHB20 element.

The well-known pull-out test for an open-ended cylinder is considered in this section. As illustrated in Figure 14a, the cylinder is pulled by two opposite radial forces, which results in the deformed shape shown in Figure 14b. The isotropic material case as well as the laminated composite material case have been considered by many authors in the literature (see, e.g., [29,30,32]). The Poisson ratio of the cylinder is ν=0.3, while the Young modulus of the metal and ceramic constituents are $E_{m}=0.7\;\times \;10^{9}\;\mathrm{Pa}$ and $E_{c}=1.51\;\times \;10^{9}\;\mathrm{Pa}$, respectively. Owing to the symmetry of the problem, only one eighth of the cylinder is modeled. The force–radial displacement curves at points A, B and C (as depicted in Figure 14a), obtained with the SHB elements, are shown in Figure 15, Figure 16 and Figure 17, respectively, along with the reference solutions taken from [13]. It can be observed that the developed SHB elements successfully pass this benchmark test as compared to the reference solutions. More specifically, the transition zone in the load–radial displacement curves, which is marked by the snap-through point, is well reproduced by both prismatic and hexahedral SHB elements for the various values of the power-law exponent *n*. Note that the converged solutions in Figure 15, Figure 16 and Figure 17 are obtained with the proposed elements by using only five integration points in the thickness direction, and meshes of (24 × 36 × 2) × 1 and 12 × 18 × 1 in the case of the prismatic SHB15 element and hexahedral SHB20 element, respectively. Hence, the required meshes for convergence are coarser than those used by Sze et al. [29] in the case of an isotropic material.

### 3.6. Pinched Hemispherical Shell

**Figure 18:**
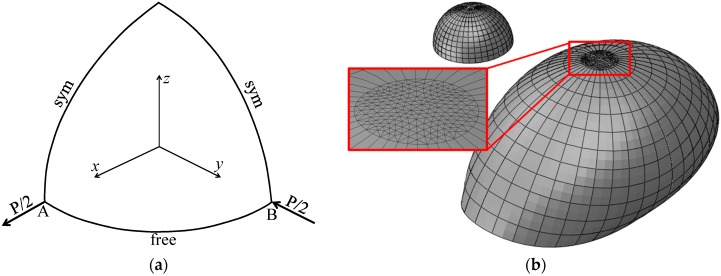
Pinched hemisphere: (**a**) geometry and (**b**) undeformed and deformed configurations.

It is worth noting that although the performance of the prismatic SHB15 element is similar to that of the hexahedral SHB20 element, as demonstrated in the above nonlinear benchmark problems, the main motivation in developing the prismatic solid–shell element is to use it for the mesh discretization of complex geometries. Indeed, it is well-known that complex geometries cannot be discretized with only hexahedral elements, and require either an irregular mesh with prismatic elements, or a mixture based on a combination of prismatic and hexahedral elements. In this section, a hemispherical shell is loaded by alternating radial forces as shown in Figure 18a. Note that this benchmark problem has been considered in the literature for an isotropic material as well as a laminated composite material (see, e.g., [30]), while the case of FGMs has not been considered yet. Consequently, only the simulation results obtained with the proposed SHB elements corresponding to the fully metallic shell can be compared to the reference solution taken from [30].

The radius and thickness of the hemispherical shell are R=10 m and t=0.04 m, respectively. The Poisson ratio of the hemispherical shell is ν=0.3, while the Young modulus of the metal and ceramic constituents are $E_{m}=6.825\times 10^{7}\;\mathrm{Pa}$ and $E_{c}=1.46\times 10^{8}\;\mathrm{Pa}$, respectively. Due to the symmetry, a quarter of the structure is discretized. The hemispherical shell is discretized with a mixture of prismatic and hexahedral elements, which consists of 90 SHB15 elements located at the top of the hemisphere (far from the load points, see Figure 18b) and 110 SHB20 elements for the remaining area.

**Figure 19:**
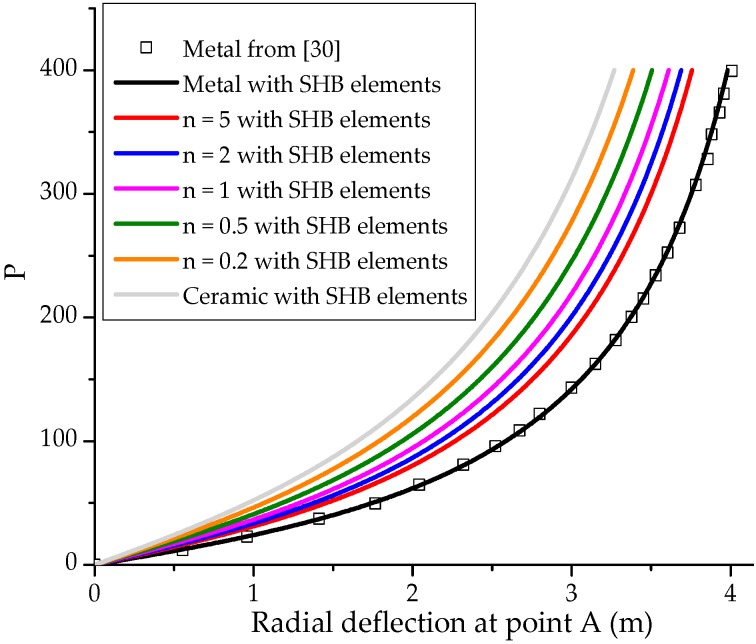
Load–displacement curves at point A for the pinched hemispherical shell, obtained with a mixture of prismatic SHB15 and hexahedral SHB20 elements.

The simulation results in terms of force–radial deflection at point A, for various values of the power-law exponent *n*, are plotted in Figure 19. This figure shows that the results corresponding to a fully metallic shell (i.e., n→∞), obtained by the combination of prismatic and hexahedral SHB elements, are in excellent agreement with those provided in [30] for an isotropic shell. Note that an equivalent in-plane mesh discretization has been used in [30], where a fully integrated shell element with several integration points has been considered. Moreover, Figure 19 reveals that for all values of the exponent *n*, the simulated load–radial deflection curves lie between that of the fully ceramic shell and that of the fully metal shell, which is consistent with the numerical results found in the previous benchmark problems.

## 4. Conclusions

In this work, quadratic prismatic and hexahedral solid–shell SHB elements have been proposed for the 3D modeling of thin FGM structures. The formulation of the SHB elements adopts the in-plane reduced-integration technique along with the assumed-strain method to alleviate various locking phenomena. A local (element) frame has been defined for each element, in which the thickness direction is specified. In this local frame, elastic properties of the thin structure are assumed to vary gradually through the thickness according to a power-law volume fraction distribution. The resulting formulations are implemented into the finite element software ABAQUS/Standard in the framework of large displacements and rotations. A series of selective and representative benchmark problems, involving FGM thin structures, has been performed to evaluate the performance of the SHB elements in geometrically nonlinear analysis. The results obtained with the SHB elements have been compared with reference solutions. Note that the state-of-the-art ABAQUS solid and shell elements have not been considered in the simulations, because these elements do not allow modeling of FGM behavior with only a single layer of elements. Overall, the numerical results obtained with the SHB elements showed excellent agreement with the available reference solutions. More specifically, the load–displacement curves for each benchmark test lie between that of the fully ceramic and fully metal behavior, which is consistent with the power-law distribution of the Young modulus in the thickness direction of the FGM plates. This good performance of the proposed SHB elements only requires a few integration points in the thickness direction (i.e., only five integration points), as compared to the number of integration points used in the literature to model thin FGM structures. Furthermore, it has been shown that the prismatic SHB15 element can be naturally combined with the hexahedral SHB20 element, within the same simulation, to help discretize complex geometries. Overall, the proposed SHB elements showed good capabilities in 3D modeling of thin FGM structures with only a single layer of elements and few integration points, which is not the case of traditional solid and shell elements.
